# A Structural In Silico Analysis of Novel Epitopes from *Toxoplasma gondii* Proteins for the Serodiagnosis of Toxoplasmosis

**DOI:** 10.3390/ijms26104689

**Published:** 2025-05-14

**Authors:** Angelis del Valle Benitez Betancourt, Tamires Lopes Silva, Débora Karolla de Freitas Oliveira, Nilson Nicolau-Junior, João Luis Garcia, Ricardo Toshio Fujiwara, Tiago Wilson Patriarca Mineo, José Roberto Mineo

**Affiliations:** 1 Laboratory of Immunoparasitology Dr. Mario Endsfeldz Camargo, Institute of Biomedical Sciences, Federal University of Uberlandia, Uberlândia 38400-902, MG, Brazil; anbbetancourt@ufu.br (A.d.V.B.B.); tlopes_s@yahoo.com.br (T.L.S.); debora.karolla@ufu.br (D.K.d.F.O.); tiago.mineo@ufu.br (T.W.P.M.); 2 Laboratory of Molecular Modeling, Institute of Biotechnology, Federal University of Uberlandia, Uberlândia 38400-902, MG, Brazil; nicolaujr@ufu.br; 3 Department of Preventive Veterinary Medicine, State University of Londrina, Londrina 86057-970, PR, Brazil; jlgarcia@uel.br; 4 Laboratory of Genomics of Parasites, Department of Parasitology, Federal University of Minas Gerais, Belo Horizonte 31270-901, MG, Brazil; fujiwara@icb.ufmg.br

**Keywords:** *Toxoplasma gondii*, toxoplasmosis, immunodominant components, in silico analysis, immunodiagnosis

## Abstract

Toxoplasmosis is a widely spread zoonosis worldwide, considered one of the most important parasitic infections that affect global public health, and usually, it is not correctly diagnosed. Serological tests for the diagnosis of *Toxoplasma gondii* infection have limitations in differentiating acute from chronic infection, which is important to determine the appropriate clinical management and treatment, mainly in pregnant women and immunocompromised individuals infected by this parasite. The present study aimed to characterize immunogenic epitopes from *T. gondii* immunodominant antigens, as SAG1(SRS29B), SAG2A (SRS34A), GRA1, GRA2, GRA3, GRA5, GRA6, GRA7, MAG1, BSR4, and CCp5A, by investigating if these parasite components might emerge as alternatives to improve the diagnosis of toxoplasmosis. A detailed comparative in silico analysis was used for this purpose. Once the protein sequences were retrieved from the ToxoDB database, different parameters were calculated, including physicochemical characteristics, accessibility values, and antigenicity. Multiple sequence alignment, 3D structures modeling, and the validation of 3D structures were also performed among all 11 peptides. Considering the results from the combination of all parameters analyzed, it can be hypothesized that the linear epitopes from SAG1, GRA3, and BSR4 proteins were found to be stable and hydrophilic, with a significant antigenicity score, and accessibility on the protein surface. Also, these three selected peptides were able to detect anti-*T. gondii* antibodies in serum samples from pigs infected by tachyzoites, when compared with control serum samples, obtained from the same naïve animals and tested by ELISA, demonstrating remarkable difference in terms of reactivity. Taken together, as our study addresses a critical challenge in the serodiagnosis of toxoplasmosis, particularly in gestational and congenital infections, where false-positive and false-negative results often arise from the use of native or recombinant antigens of *T. gondii*, our findings highlight the potential of synthetic peptides derived from novel epitopes of this parasite as alternative tools for the development of more accurate immunodiagnostic assays for toxoplasmosis.

## 1. Introduction

Toxoplasmosis is a widely spread zoonosis worldwide, caused by the obligate intracellular parasite *Toxoplasma gondii*, a protozoan with the ability to infect a large number of warm-blooded animal species, including humans [1,2]. With approximately one-third of the world’s population chronically infected by different genotypes, and a prevalence that varies extensively depending on the geographic region, toxoplasmosis is one of the neglected parasitic infections requiring active health control [3,4,5,6]. In this regard, the development of effective diagnostic methods, as well as vaccines, is necessary to avoid the consequences of toxoplasmosis on global public health and the economy [7].

Usually, infection is asymptomatic in immunocompetent individuals, sometimes causing nonspecific clinical signs [8]. From the medical point of view, the recognition of the infection is relevant in pregnant women, where the risk of transmission of the parasite to the fetus is high, and the associated consequences can compromise the life of the fetus and infected mothers. The detection of the parasite is also important in immunosuppressed individuals, such as HIV, cancer, and transplant patients, where the reactivation of the disease can cause severe health problems, which depending on various factors can even lead to the death of these patients [9,10].

The diagnosis of *T. gondii* infection through sensitive and specific methods is a fundamental step in treating and managing patients with suspected toxoplasmosis. *T. gondii* is capable of inducing a potent immune response that generates persistent antibody titers; thus, the diagnosis of toxoplasmosis carried out by serological tests, with emphasis on the detection of specific IgA, IgM, and IgG antibodies, against antigens of the parasite is useful [11,12]. While humans can be infected by any of the three infectious stages (sporozoites, bradyzoites, and tachyzoites), most commercial tests use native antigens derived from *T. gondii* tachyzoites. Despite their high sensitivity and specificity, it has been shown that the use of these antigens has limitations associated with different production and purification methods, laborious procedures, and difficulties in standardizing which finally cause ambiguous results, avoiding the accurate diagnosis and effective therapeutic and preventive procedures [11,13]. Moreover, it has been described in the literature that these antigenic preparations lack the ability to differentiate the clinical phases of infection (acute and chronic) [14,15].

Due to the difficulties associated with using native *T. gondii* antigens, new diagnostic tools are being investigated. Recombinant antigens, chimeric antigens, and peptides derived from *T. gondii* proteins have been suggested by various studies, not only to improve the diagnosis of *T. gondii* infection but also to improve the ways to discriminate the different stages of toxoplasmosis [11,12,16,17,18]. The use of bioinformatics tools, for the characterization of these new antigenic peptides capable of selectively stimulating the B-cell response, has a relevant role in making it possible to understand the antigenic structure of pathogens and to design new diagnostic methods and vaccine applications for several diseases [19].

The life cycle of *T. gondii* is complex and the expression of its proteins changes during different stages of development, some of which have been recognized to activate the immune system, generating antibody titers that remain throughout the life of the host [20,21]. The surface antigens SAG1 and SAG2A of *T. gondii* are highly immunogenic proteins that are expressed primarily on tachyzoites [22,23]. SAG1 has been used in several serodiagnosis studies that have suggested the usefulness of this antigen in detecting IgG antibodies in chronically infected individuals [24,25]. SAG2A has been considered a potential marker for the diagnosis of acute toxoplasmosis in humans [26].

Dense granule proteins (GRA) play an important role in the process of host-cell invasion. They have shown good antigenicity, expressed by both tachyzoites and bradyzoites, and they have been recognized as both acute and chronic phase markers [7,27,28,29]. The GRA 1, 2, 3, 5, 6, and 7 antigens have demonstrated utility in the detection of antibodies to *T. gondii* with high sensitivity in acute and chronic phases of toxoplasmosis [27,30,31,32,33].

The surface antigen BSR4 and matrix antigen 1 (MAG1) are bradyzoite-specific proteins involved in human B and T cell responses [34,35]. In an ELISA assay, BSR4 protein demonstrated specific immunoreactivity in sera from patients in the chronic phase and MAG1 detected antibodies in the acute stage more frequently than in the chronic stage of toxoplasmosis [34,36]. CCp5A is a polypeptide specifically expressed by *T. gondii* sporozoites and is considered a good molecular marker to differentiate infectious stages [18]. Thus, the identification of B-cell epitopes from *T. gondii* immunodominant proteins could be a determining factor in the diagnosis of *T. gondii* infection and differentiation of clinical stages, from the appropriate selection of epitopes of antigens expressed in different stages of the parasite [37].

In the present study, an in silico comparative analysis was performed to characterize B-cell epitopes from the sequence of immunodominant proteins using different bioinformatics tools, aiming to provide new alternatives to improve the diagnosis of *T. gondii* infection.

## 2. Results

### 2.1. B-Cell Epitope Prediction Design

**Figure 1:**
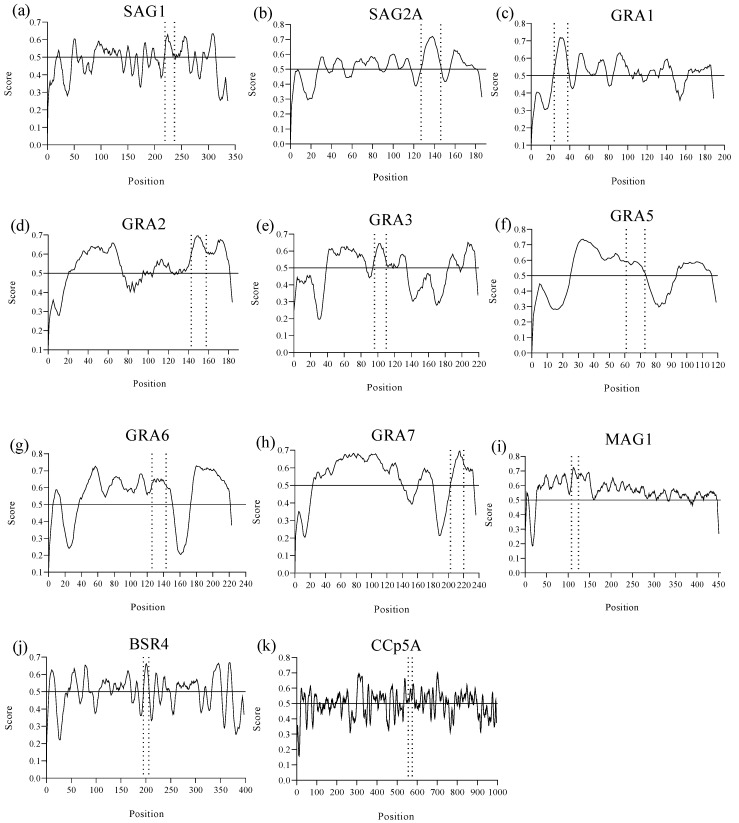
Epitope prediction from *T. gondii* immunodominant proteins by BepiPred Linear Epitope Prediction version 2.0 from the Immune Epitope Database and Analysis Resource (IEDB) (https://www.iedb.org/, accessed on 3 April 2025) to identify the presence of linear B-cell epitopes. The figure demonstrates the predicted peptides delimited by the dashed lines that showed the best scores (≥0.5) arranged in the least polymorphic regions of the following *T. gondii* proteins: (**a**) SAG1, (**b**) SAG2A, (**c**) GRA1, (**d**) GRA2, (**e**) GRA3, (**f**) GRA5, (**g**) GRA6, (**h**) GRA7, (**i**) MAG1, (**j**) BSR4, (**k**) CCp5A.

Using reference scores equal to or greater than 0.5 in each prediction, as suggested in the BepiPred 2.0 method, it was possible to identify a total of 11 linear B-cell epitopes from the SAG1, SAG2A, GRA1, GRA2, GRA3, GRA5, GRA6, GRA7, MAG1, BSR4, and CCp5A *T. gondii* immunodominant proteins, with amino acid sequences between 12 and 21 residues, arranged in the least polymorphic regions of the proteins and with the best scores, as shown in Figure 1. The numbers of predicted epitopes, their length, position, as well as the protein to which they belong, are shown in Table 1.

### 2.2. Homology and Phylogenetic Relationship

**Figure 2:**
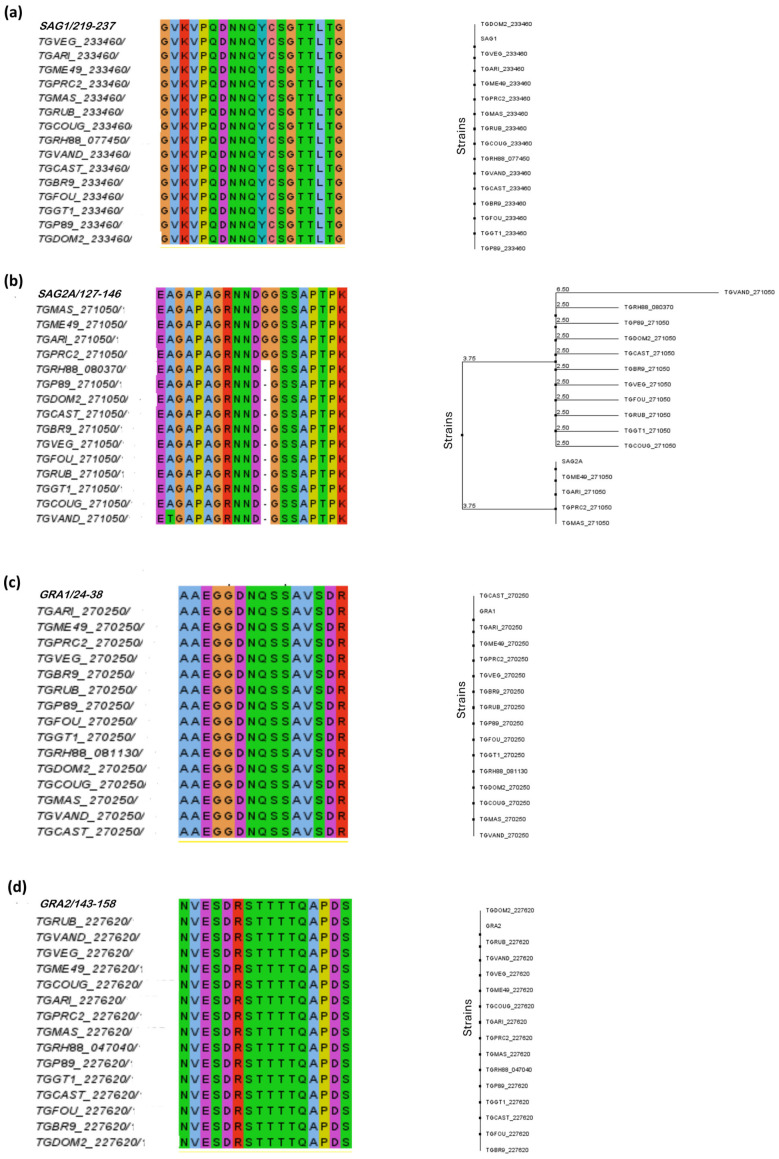

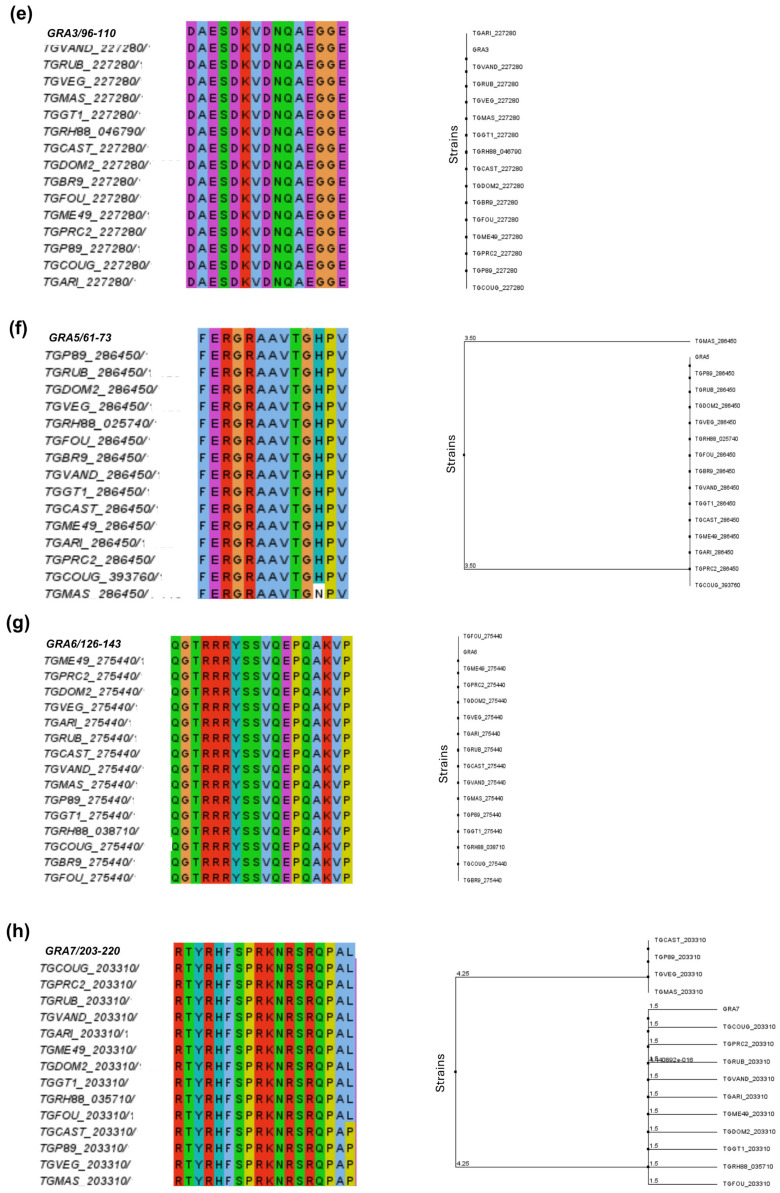

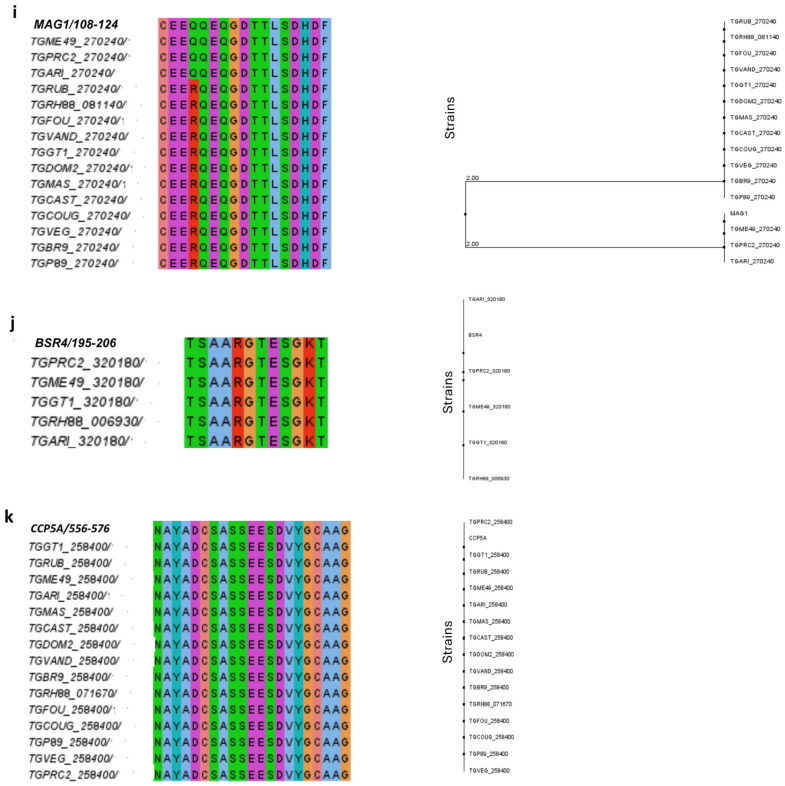
A phylogenetic analysis of predicted epitopes from *T. gondii* immunodominant proteins. The figure shows the multiple sequence alignment for each predicted epitope and the respective phylogenetic trees designed in Jalview version 2 platform. The different colors in the alignment refer to the polarity of the amino acids in the sequences of the peptides. Note that the alignment of most peptides was possible in 15 *T. gondii* strains, except for the BSR4 peptide, where alignment was possible with only 5 strains. The phylogenetic alignment and phylogenetic trees are shown in the following order: (**a**) SAG1, (**b**) SAG2A, (**c**) GRA1, (**d**) GRA2, (**e**) GRA3, (**f**) GRA5, (**g**) GRA6, (**h**) GRA7, (**i**) MAG1, (**j**) BSR4, (**k**) CCp5A.

To verify that the predicted peptides were conserved among the *T. gondii* strains of interest, multiplex sequence alignment was performed. The results of alignment and phylogenetic tree construction are shown in Figure 2.

According to the alignment, it was possible to observe that the predicted peptides for SAG1, GRA1, GRA2, GRA3, GRA6, and CCp5A proteins showed the highest degree of conservation and identity in the different *T. gondii* strains, such as types I, II, and III of epidemiological importance.

Regarding the SAG2A peptide, it was possible to observe that most residues were highly conserved, with good alignment quality and consensus among amino acids; however, it is known that the SAG2A protein is highly polymorphic. Only the addition of glycine (G) residue was present in strains ME49, MAS, ARI, and PRC2, with a consensus of 31%. It was not present in type I and type III strains. The substitution of the amino acid alanine (A) for threonine (T) at position 128 was observed in *T. gondii* strain VAND (Figure 2b).

For GRA5, most residues were conserved, and a conservative substitution of histidine (H) by an asparagine (N) was observed at position 71 in the *T. gondii* MAS strain (Figure 2f). After analyzing the GRA7 peptide, was noticed a substitution of leucine (L) by a proline (P) in the strains CAST, P89, VEG, and MAS (Figure 2h)

The sequence alignment of MAG1 resulted in a high percentage of identity and similarity, but the strains ME49, GARI, and PRC2 showed a non-conservative substitution of the amino acid arginine (R) by glutamine (Q) at position 111 (Figure 2i). The BSR4 peptide was totally conserved only in the 5 strains for which the protein has been described, among them the RH and ME49 strains (Figure 2j).

### 2.3. General Characteristics of Peptides: Physicochemical Properties, Accessibility, and Antigenicity

The physicochemical parameters calculated using Expasy’s ProtParam tool are shown in Table 2. The server results showed that all peptides had a molecular weight above 1000 kDa. For most peptides, the isoelectric point values were less than 7 and were considered to be acidic. In this case, the predicted peptides would be more stable at acidic pH, except for peptides GRA5, GRA6, GRA7, and BSR4, which would be more stable at basic pH.

Defined as the relative volume occupied by aliphatic side chains (alanine, valine, isoleucine, and leucine), the aliphatic index provides an estimate of the thermal stability of a protein. The aliphatic index of the peptides ranged from 16.67 to 60.00; the higher the aliphatic index, the higher the probability that the peptides are more stable over a wide temperature range. The predicted peptides showed an acceptable aliphatic index, with the GRA5 peptide having the highest index, and were thus considered to be thermostable.

The instability index estimates the stability of the protein in a test tube. A protein with an instability index lower than 40 is considered stable, while a value higher than 40 predicts that the protein may be unstable. SAG1, GRA3, GRA5, and BSR4 peptides were more stable when compared to the SAG2A, GRA1, GRA2, GRA6, GRA7, MAG1, and CCp5A peptides.

Regarding the hydropathicity index (GRAVY), the calculated values for the peptides were between −1.713 and −0.281. Negative values indicate better interaction with water, and greater solubility, that is, more hydrophilic peptides, while positive GRAVY values indicate greater hydrophobicity.

**Figure 3:**
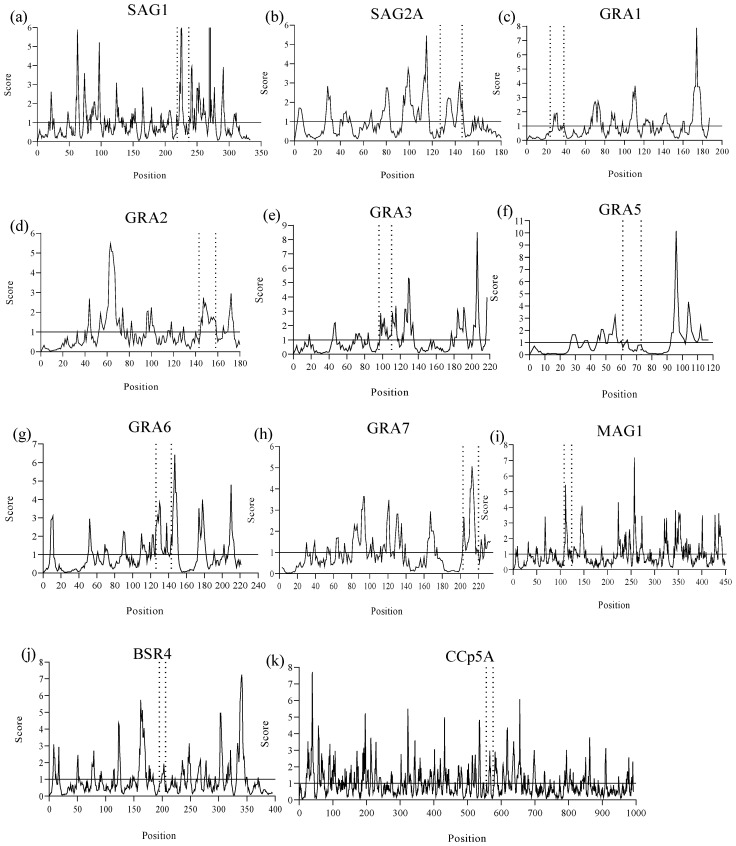
An analysis of surface accessibility of the predicted epitopes using Emini surface accessibility prediction tool of Immune Epitope Database and Analysis Resource (IEDB) (https://www.iedb.org/, accessed on 3 April 2025). The image shows the predicted peptides delimited by the dashed lines and their amino acid residues that hold the default threshold value of 1.0, indicating the probability of these residues being found on the surface of their respective *T. gondii* proteins: (**a**) SAG1, (**b**) SAG2A, (**c**) GRA1, (**d**) GRA2, (**e**) GRA3, (**f**) GRA5, (**g**) GRA6, (**h**) GRA7, (**i**) MAG1, (**j**) BSR4, (**k**) CCp5A.

Using the Emini method, the solvent accessibility of epitopes was evaluated. It was observed that when the amino acid residues had a score equal to or greater than the 1.0 threshold, the probability of this peptide being found on the protein surface increased (Figure 3). In this sense, peptides from SAG1, GRA2, GRA3, GRA6, GRA7, MAG1, and BSR4 were found in more accessible regions within the protein structures, whereas SAG2A, GRA1, GRA5, and CCp5A peptides were less exposed with only a few solvent-accessible residues, making it difficult for them to interact with water molecules.

Using the VaxiJen 2.0 server, the antigenic potential of each predicted linear B-cell epitope was analyzed. The results showed that the epitopes had antigenicity values ranging from 0.3953 to 1.4757, with those that demonstrated values above the threshold of 0.5 being considered antigenic epitopes. All selected peptides were considered as probable antigens, except for the GRA6 peptide that showed the lowest score according to the server. Table 3 demonstrates the antigenicity values for each epitope.

### 2.4. Three-Dimensional Modeling of Proteins and Validation

The three-dimensional structures of 10 *T. gondii* proteins were not yet available in PDB format. Therefore, the 3D structures for the proteins SAG2A, GRA1, GRA2, GRA3, GRA5, GRA6, GRA7, MAG1, BSR4, and CCp5A were modeled using the Robetta server. The 3D structure for SAG1 was not modeled because it was available in PDB format.

In terms of stereochemical quality, the predicted models were analyzed using the Ramachandran plot. The results of the Ramachandran plots are presented in Table S1 and Figure S1, showing the phi–psi torsion angles for all residues in the 3D structure of proteins. Generally, it is expected to have at least 90% of the residues in the most allowed regions of phi–psi values. All the models constructed presented more than 90% of their amino acid residues between the most allowed and additional allowed regions. Although the proteins SAG2A, GRA2, GRA3, GRA6, GRA7, MAG1, and CCp5A presented amino acid residues between the non-allowed zones, the combination of the Ramachandran and G-factor data suggests an overall good quality of the models.

The results of the ERRAT and PROVE tools are shown in Table 4. The overall quality factor of the modeled proteins resulted in values of 89.4467–100, suggesting models with adequate resolutions. The PROVE values were between 0.8 and 4.2%.

### 2.5. Three-Dimensional Modeling of Peptides and Validation

**Figure 4:**
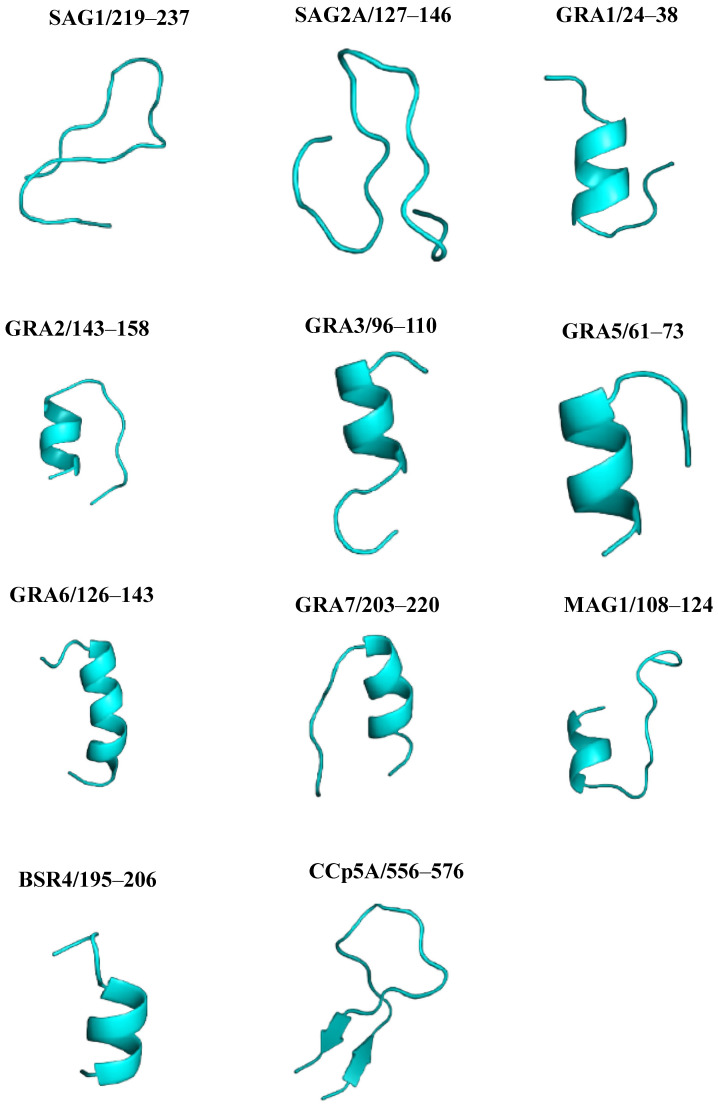
*T. gondii* peptide structure prediction and analysis using the Pep Fold server (https://bioserv.rpbs.univ-paris-diderot.fr/services/PEP-FOLD/, accessed on 3 April 2025). The figure shows the best three-dimensional structures (blue) of peptides derived from *T. gondii* immunodominant proteins.

All 11 linear B-cell epitopes predicted were modeled using the Pep fold server. From the amino acid sequence, the site predicts a consistent peptide structure on a large scale, providing a total of 5 models for each of the epitopes, the best of which were selected. The generated 3D models are shown in Figure 4.

After analyzing the Ramachandran plot and G-factors, amino acid residues were arranged in both the most optimal and additional permitted regions of the diagram. These parameters suggest that the 3D structure of the peptides is adequate, stable, and of good stereochemical quality (Table S2) (Figure S2).

The overall quality factor of all modeled peptides was 100, indicating that the models predicted here exhibit good high resolution. The z-score for most peptides demonstrates lower atomic values. The ERRAT and PROVE analyses of peptide three-dimensional modeled structures are shown in Table 4.

### 2.6. PYMOL Alignment

**Figure 5:**
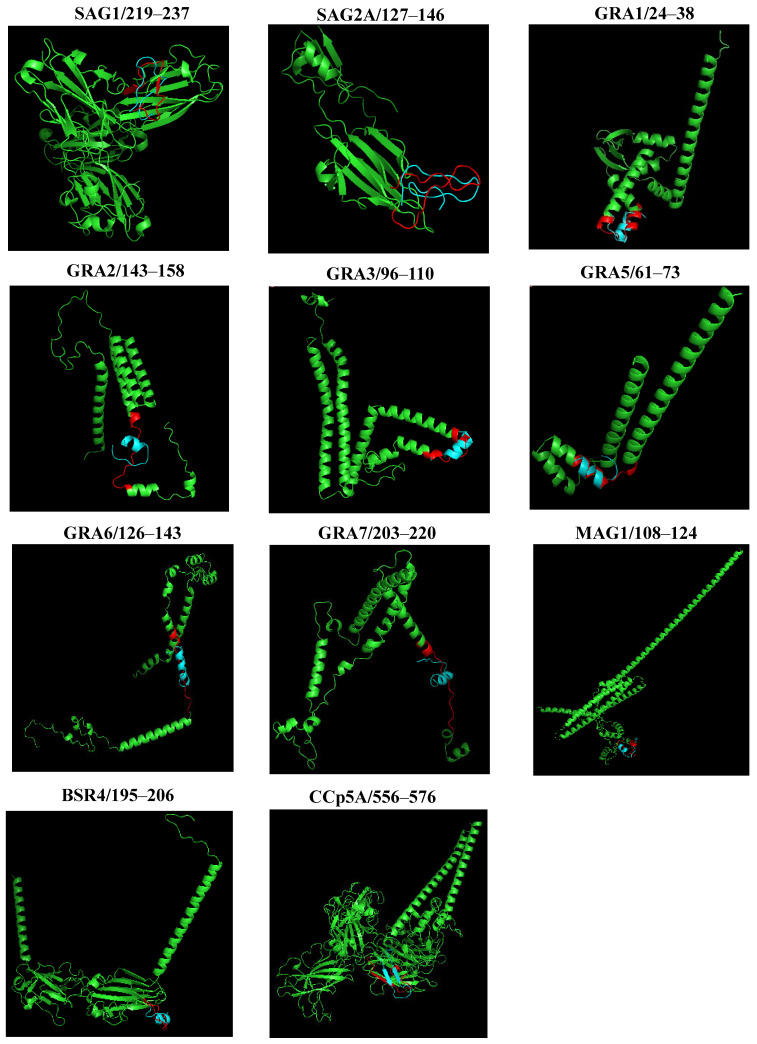
A structural representation of the alignment of the 3D structures of proteins and peptides obtained through PYMOL. The colors were used to indicate the 3D structure of the *T. gondii* proteins (green), the peptide region (red), and the 3D structures of the aligned peptides (blue) by PYMOL.

To compare the predicted structures and visualize the arrangement of peptides in the proteins, a structural alignment was performed. Initially, the PYMOL server performs the sequence alignment and then the structure alignment, calculating the RMSD. Figure 5 shows the graphical representation of the structural alignment made in the PYMOL program. It can be seen that the peptides showed differences in structural conformation and accessibility on the surface protein. The secondary structure analysis suggested that peptides from SAG1, and SAG2A, presented a more distended disposition forming random coils, while peptides GRA1, GRA2, GRA3, GRA5, GRA6, GRA7, MAG1, BSR4, and CCp5A adopted folding conformations, forming an α-helix and, in the case of CCp5A, forming a β-hairpin in their respective proteins.

### 2.7. Biological Activity of the Selected Peptides

**Figure 6:**
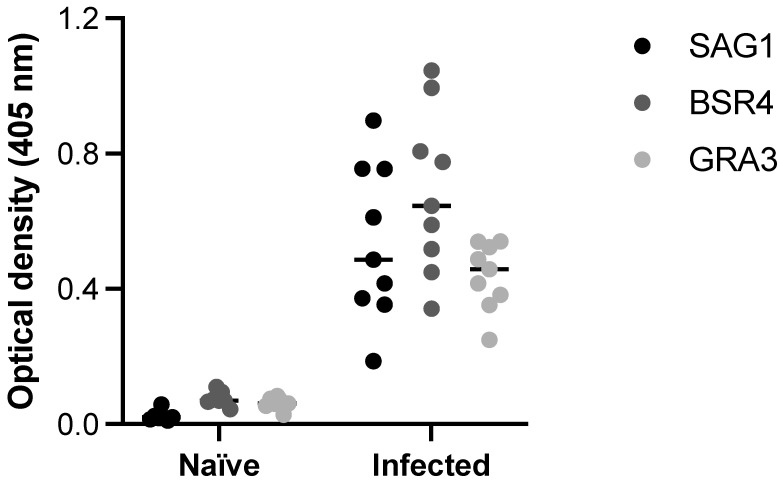
ELISA with SAG1, BSR4, and GRA3 peptides as antigenic preparations. The detection of anti-*T. gondii* antibodies in serum samples from pigs infected by tachyzoites, compared with control serum samples (naïve), obtained from the same animals before infection. The reactivity using the three selected peptides was determined by optical densities at 405 nm.

As shown in Figure 6, ELISA carried out with SAG1, BSR4 and GRA3 peptides was able to detect anti-*T. gondii* antibodies in serum samples from pigs infected by tachyzoites. When compared with control serum samples, obtained from the same animals before infection (naïve), samples from infected animals tested by ELISA using the three selected peptides demonstrated remarkable difference in terms of reactivity. When the ELISA peptides were compared with the reference test (STAg ELISA) to obtain the co-positivity (sensitivity) and co-negativity (specificity) scores, the results demonstrated 100% agreement for both immunoassays.

## 3. Discussion

The sensitivity and specificity of immunodiagnostic assays for toxoplasmosis depend on the antigens used [37]. New alternative immunoassays have been proposed in the literature to replace the use of native *T. gondii* antigens, aiming to solve the limitations related to production cost and standardization of those antigenic preparations and making possible to obtain significant improvements in the detection of phase of infection by this parasite [11].

Advances in methodologies to identify epitopes by combining computational methods and mathematical algorithms using an extensive biological database have contributed to the development of bioinformatics. This novel tool makes it possible to analyze the structure of antibodies, B cells, and T cells, evaluate intermolecular interactions, characterize and model immunogenic epitopes, and design new vaccines and diagnostic methods [38]. Synthetic peptides predicted using bioinformatics tools offer distinct advantages such as shorter production time and cost when compared to lysed *T. gondii* antigens, accurate knowledge of antigen composition with a high degree of purity, and ease of method standardization [16,39]. Using a set of bioinformatics web servers, a group of 11 peptides from the antigenic components of *T. gondii* was assessed, as follows: SAG1, SAG2A, GRA1, GRA2, GRA3, GRA5, GRA6, GRA7, MAG1, BSR4, and CCp5A, which are expressed in the different infectious stages of the parasite, as sporozoites, tachyzoites, and bradyzoites, as previously characterized [7,18,22,23,27,28,29,35].

When the whole data were analyzed and compared, peptides predicted from SAG1, GRA3, and BSR4 proteins, in particular, showed superior physicochemical, accessibility, and antigenic properties. The theoretical isoelectric point for these peptides was 5.83, 3.71, and 8.41, respectively. The isoelectric point indicates the pH at which a protein has zero net charges. At this pH there is a decrease in repulsive interactions and, therefore, the interaction between the molecules is improved; thus, even the proteins are more stable [40]. In that sense, the stability and solubility of these peptides is maintained between acidic and basic pH. The calculated isoelectric point will be useful for determining the solutions and buffer system needed to establish conditions for the standardization and development of new designed immunoassays. In terms of aliphatic index and instability index values, the peptides were stable and moderately thermotolerant, with high hydrophilicity due to negative GRAVY values, which is one of the important characteristics of antigenic epitopes that allow for the understanding the protein folding, predicting the secondary structure and interaction sites [7,41].

The peptides from the SAG1, GRA3, and BSR4 proteins presented significant antigenicity values of 1.1163, 1.2841, 1.4757, respectively. In addition, the multiple sequence alignment demonstrated that the peptides selected from SAG1, GRA3, and BSR4 proteins showed a high degree of conservation and identity in different *T. gondii* strains, such as the epidemiologically important type I, II, and III strains [42]. Also, a BLASTp search using these specified epitope sequences was performed against protozoan parasite databases, revealing that all of them are highly conserved within *T. gondii* strains and do not have homologs in other protozoan parasites, including *Neospora caninum*, *Plasmodium* sp., *Leishmania* sp., *Trypanosoma* sp., among others. This suggests that the chosen epitopes are appropriate, which is advantageous for developing species-specific diagnostic tools. It is also necessary to consider that, although a BLASTp search is useful for demonstrating specificity, the occurrence of cross-reactions cannot be excluded.

Approximately 30% of the human population is infected by various widely distributed genotypes of *T. gondii*, with clonal lineages type I, II, and III being the most important [6]. In addition to the classical clonal lineages, further genetic diversity of *T. gondii* associated with other divergent lineages, called atypical strains, has been described [42]. The classical clonal lineages represent a large majority of isolates in North America and Europe, with the type II lineage being the most predominant in these continents and the rest of the world. In South America, *T. gondii* strains show great genetic variability, with unique polymorphisms [42]. The identification of significantly antigenic epitopes that are also conserved among the different strains of *T. gondii* could aid the diagnosis of toxoplasmosis by increasing case detection independently of the infecting strain.

The prediction of the 3D models and the validation of the structure by Ramachandran plot analysis revealed that these peptides had more than 90% of their residues in energetically favorable regions of the diagram. In addition, the overall quality factor and PROVE of all modeled peptides mean that the quality of the modeling has been satisfactory. Thus, most of the amino acid residues for these peptides were found exposed on the surface of the proteins, adopting distinct structural conformations, which facilitate their interaction with molecules of the immune system. Secondary structures are of great importance for the epitopes, as the α-helices and β-turns have high-energy hydrogen bonds that maintain the structural conformation of the protein and therefore strengthen interactions with antibodies [43].

Numerous bioinformatics tools have been useful in the identification of immunogenic epitopes of various *T. gondii* antigens that may have better potential to ensure an accurate diagnosis [44]. SAG1 is known to be one of the most immunogenic *T. gondii* antigens and is considered a promising molecule for the development of diagnostic methods and vaccines by eliciting a strong immunodominant response [45]. In this sense, the reactivity of SAG1-derived epitopes has been tested with different clinical forms of toxoplasmosis in some studies, suggesting the potential for detecting infected individuals [46]. GRA3 has been used in association with the most immunoreactive regions of other *T. gondii* proteins, demonstrating a relevant performance in serodiagnosis [27].

BSR4 protein has demonstrated immunogenicity and protein-specific immunoreactivity in sera from patients in the chronic phase in ELISA assay; however, the diagnostic utility of peptides derived from this protein for the serodiagnosis of toxoplasmosis has not been described, so it would be interesting to further investigate its potential for detecting *T. gondii* infection [34].

The characterization and use of new immunodominant epitopes from these proteins would allow us to formulate strategies to improve the diagnosis of *T. gondii* infection. Studies suggest the use of chimeric antigens for ELISA, based on multiplex antigenic epitopes expressed at different stages of the parasite, which should be recognized by antibodies against individual antigens and increase the sensitivity and specificity of diagnostic tests for toxoplasmosis. [47]. Using an in silico approach, Beghetto et al. (2006) [27] designed a multi-epitope with sequences of six immunodominant *T. gondii* proteins, among which SAG1 and GRA3 were present. The study indicated the significant diagnostic performance of the chimeric antigen in detecting acquired infection in adults and in infants born to mothers with a primary *T. gondii* infection. Holec-Gąsior et al. (2012) [48], using peptides of MIC1, MAG1, and SAG1 bound in a chimera, obtained significant results when compared to the multi-epitope with just MIC1 and MAG1 sequences, suggesting the immunogenicity of SAG1. The use of individual recombinant or chimeric formulations has shown the potential to differentiate *T. gondii*-infected individuals from uninfected individuals, as well as the stage of infection [11,12,49,50].

The use of chimerical antigens in the diagnosis of infections offers a high density of immunoreactive epitopes of various antigens that increase the chances of detection of antibodies in serum samples and, therefore, improve the performance of the immunoassay [51]. The in silico analysis performed here, therefore, suggests that the peptides from SAG1, GRA3, and BSR4 protein could be used individually or together in new immunodiagnostic assays to validate their potential in detecting specific anti-*T. gondii* antibodies.

Overall, this study highlights the physicochemical properties, accessibility, and antigenicity of three synthetic peptides, selected based on the prediction and analysis of linear B-cell epitopes from the protozoan parasite Toxoplasma gondii. However, it is important to acknowledge the limitations of the study, as peptide selection was based solely on the highest-scoring candidates identified through bioinformatic tools, out of a total of eleven predicted peptides. Future research should include an experimental evaluation of the remaining eight epitopes, and extend beyond those derived from SAG1, BSR4, and GRA3. In this context, additional ELISA assays should be conducted using all characterized epitopes, with performance compared to that of the STAg-based ELISA.

## 4. Materials and Methods

### 4.1. Prediction and Analysis of Linear B-Cell Epitopes

To analyze the presence of linear B-cell epitopes in *T. gondii* immunodominant antigens, complete amino acid sequences of the proteins SAG1, SAG2A, GRA1, GRA2, GRA3, GRA5, GRA6, GRA7, MAG1, BSR4, and CCp5A were obtained in FASTA format from the ToxoDB database (https://toxodb.org/toxo/app, accessed on 3 April 2025). The sequences of each protein were thoroughly analyzed using bioinformatics tools for immunological epitope prediction, available online in the Immune Epitope Database and Analysis Resource (IEDB) software (https://www.iedb.org/, accessed on 3 April 2025). The prediction of linear epitopes was made based on data obtained from the BepiPred Linear Epitope Prediction version 2.0 method (BepiPred-2.0: Sequential B-Cell Epitope Predictor), using reference scores equal to or greater than 0.5. Using the Random Forest Regression algorithm, the BepiPred-2.0 server is able to predict linear B-cell epitopes from the 3D structure of the protein. BepiPred-2.0 represents a method with improved predictive power compared to other methods available online, which predicts that residues with values above the threshold (predetermined value 0.5) are part of a B-cell epitope [52].

### 4.2. Sequence Alignment and Estimation of Phylogenetic Trees

The multiple sequence alignment of *T. gondii* immunodominant proteins was carried out on the MUSCLE website (https://www.ebi.ac.uk/Tools/msa/muscle/, accessed on 3 April 2025). The obtained information was used to analyze and evaluate the homology and conservation of the chosen peptides among *T. gondii* strains, and to estimate phylogenetic trees using the Jalview version 2 platform (https://www.jalview.org/, accessed on 3 April 2025) [53].

### 4.3. Prediction of Physicochemical Properties, Accessibility, and Antigenicity

To estimate the physicochemical properties of the selected epitopes, the web tool ProtParam (ExPASy) was used (https://web.expasy.org/protparam/, accessed on 3 April 2025), which calculated the main parameters of the sequences such as molecular weight (MW), theoretical isoelectric point (pI), aliphatic index, instability index, and average hydropathicity (GRAVY). From this analysis, it was possible to characterize the peptides.

The solvent accessibility of the selected peptides was determined using the Emini surface accessibility prediction tool [54] available on the IEDB website. The Emini method calculates the probability of amino acids being exposed on the surface of a given protein when the score is greater than the set threshold value (greater than or equal to 1).

Antigenicity forecasting of peptides was carried out through the free web server VaxiJen v2.0 (http://www.ddg-pharmfac.net/vaxijen/VaxiJen/VaxiJen.html, accessed on 3 April 2025). The prediction of antigenicity through the VaxiJen v2.0 website is based on the physicochemical characteristics of the proteins of a given organism (parasites, bacteria, fungi, viruses) and the transformation of the sequences into uniform vectors of the main amino acid properties using the automatic cross-covariance (ACC) system [55].

### 4.4. Prediction of the Three-Dimensional (3D) Model of the T. gondii Proteins

The construction of the three-dimensional structure of SAG1, SAG2A, GRA1, GRA2, GRA3, GRA5, GRA6, GRA7, MAG1, BSR4, and CCp5A proteins was performed in the Robetta server (https://robetta.bakerlab.org/, accessed on 3 April 2025). Robetta is a free online service that analyzes and predicts protein structure using the methods of comparative modeling or de novo structure prediction. Through an automated interface, sequences sent to the server are analyzed in putative domains and a protein model is predicted in the presence or absence of sequence homology with proteins of known structure. The server produces PDB extension files that enabled an analysis of the generated structures in Discovery Studio and PYMOL [56].

### 4.5. Prediction of the Three-Dimensional (3D) Model of the T. gondii Peptides

Using the Pep Fold website (https://bioserv.rpbs.univ-paris-diderot.fr/services/PEP-FOLD/, accessed on 3 April 2025) the 11 predicted peptides were modeled. For the 3D modeling of peptides of up to 52 amino acid residues, Pep Fold uses the structural alphabet (SA) concept derived from the hidden Markov model and ensembles the predicted fragments using an algorithm and modified version of the coarse-grained force field (OPEP) [57].

### 4.6. Validation of the 3D Modeling of Proteins and Peptides

In the next step, the quality of 3D models obtained using Robetta and Pep Fold software was checked by Ramachandran plot analysis of PDB SUM website (http://www.ebi.ac.uk/thornton-srv/databases/cgi-bin/pdbsum/GetPage.pl?pdbcode=index.html, accessed on 3 April 2025), ERRAT and PROVE tools of SAVES 6.0 server, also were used (https://saves.mbi.ucla.edu/, accessed on 3 April 2025).

With the Ramachandran diagram, it is possible to detect phi–psi torsion angles for all amino acid residues in a structure, allowing for an analysis of which residues lie outside the energetically favorable regions and thus verifying the conformational or stereochemical stability of the protein [58]. ERRAT validates the structure of a protein based on the interaction and distribution of the different types of atoms, which can be distinguished using a quadratic error function. Generally, good high-resolution structures produce values around 95% or higher, and lower resolutions the overall quality factor is around 91% [59]. PROVE evaluates the quality of protein structures by comparing the deviations of the atomic volumes (Z-scores) with the standard values of highly resolved and refined proteins, since better-resolved models present a lower Z-score [60].

### 4.7. Three-Dimensional Structure Alignment of Proteins and Peptides

Once modeled and validated, the 3D structures of the proteins and their respective predicted peptides were aligned using PYMOL version 3.0 software (https://pymol.org/2/, accessed on 3 April 2025). PYMOL is a tool that allowed for the visualization of the 3D models as well as analysis of the arrangement of the peptides calculating the Root Mean Square Deviation (RMSD) and their accessibility on the protein surface.

### 4.8. Serum Samples from Uninfected and Experimentally Infected Pigs

Serum samples were obtained from experimentally infected pigs, as previously described [61]. Briefly, nine 6- to 8-week-old mixed-breed pigs, seronegative for *T. gondii*, were lodged in separate stables, and received an intramuscular infection with 7 × 10^7^ tachyzoites of the RH strain. The control group was constituted by the serum samples from the same animals prior to the infection. Serum was collected weekly after the infection, and we chose samples obtained between 14 and 28 days after the inoculation to test the antigenicity of the selected peptides. All samples were checked for seroconversion by using *T. gondii* native soluble antigen [STAg] ELISA. This experimental protocol was approved by the Ethical Committee for Experimental Utilization of Animals from the State University of Londrina (CEUA 17/09).

### 4.9. ELISA to Evaluate the Biological Activity of the Selected Peptides

For ELISA using synthetic peptides, previous optimization of the reaction was established through block titration of the selected peptides, blocking buffers, serum samples, and conjugated antibodies. High-binding 96-well plates (Corning, New York City, NY, USA) were sensitized with 2 µg/well of each peptide in sodium carbonate buffer (pH 9.6) and incubated overnight, at 37 °C. The wells were washed 4 times with 100 µL of PBS-Tween 20 and blocked with 5% bovine serum albumin (BSA) in PBS, for 1 h at 37 °C. The serum samples were diluted at 1:100 in PBS in 2.5% BSA and incubated for 1 h at 37 °C. After 4 washes, anti-pig IgG peroxidase (Bio-Rad, Santo Amaro, Brazil) was used as the secondary antibody, at 1:2000 dilution in 2.5% BSA in PBS. Again, the plates were incubated 1 h at 37 °C, and after a new washing cycle, the reaction was revealed with 2.2′-azino-bis-3-ethyl-benzothiazoline sulfonic acid peroxidase substrate (ABTS, Thermo, São Paulo, Brazil) and the optical densities were read at 405 nm (M2e, Molecular Devices, San Jose, CA, USA). The results of ELISA peptide were compared as a percentage of co-positivity (sensitivity) and co-negativity (specificity) in relation to the results obtained by the reference test, STAg ELISA.

## 5. Conclusions

The current limitations of the antigens used in the immunodiagnosis of *T. gondii* infection suggest the necessity to develop new antigenic preparations, aiming to increase the accuracy of toxoplasmosis, particularly to characterize the phase of infection. Bioinformatic methods allow for the identification of immunodominant regions of a particular antigen in less time and cost. Through in silico analysis, 3 out of 11 peptides were selected from the parasite, SAG1, BSR4, and GRA3. These selected components were found to be stable, hydrophilic, and protein-surface accessible, with an antigenic role, suggesting their potential use for toxoplasmosis diagnosis as a single or multiepitope tool to design new immunodiagnostic assays.

## Figures and Tables

**Table 1:** The predicted peptides from *T. gondii* immunodominant proteins by Bepipred Linear Epitope Prediction version 2.0, characterized by their length and position in each protein.

Protein	Peptide Identification	Peptide Sequences	Length	Position
SAG1	Pep1	GVKVPQDNNQYCSGTTLTG	19	219–237
SAG2A	Pep2	EAGAPAGRNNDGGSSAPTPK	20	127–146
GRA1	Pep3	AAEGGDNQSSAVSDR	15	24–38
GRA2	Pep4	NVESDRSTTTTQAPDS	16	143–158
GRA3	Pep5	DAESDKVDNQAEGGE	15	96–110
GRA5	Pep6	FERGRAAVTGHPV	13	61–73
GRA6	Pep7	QGTRRRYSSVQEPQAKVP	18	126–143
GRA7	Pep8	RTYRHFSPRKNRSRQPAL	18	203–220
MAG1	Pep9	CEEQQEQGDTTLSDHDF	17	108–124
BSR4	Pep10	TSAARGTESGKT	12	195–206
CCp5A	Pep11	NAYADCSASSEESDVYGCAAG	21	556–576

**Table 2:** Physicochemical properties of the selected peptides calculated by Expasy’s ProtParam online server.

Peptide	Molecular Weight (Da)	Theoretic pI	Aliphatic Index	Instability Index	GRAVY
SAG1	1982.15	5.83	51.05	17.03	−0.721
SAG2A	1853.92	6.17	20.00	56.12	−1.195
GRA1	1463.44	4.03	39.33	49.64	−1.040
GRA2	1708.71	4.03	24.38	58.81	−1.425
GRA3	1563.51	3.71	32.67	−0.99	−1.713
GRA5	1396.57	9.61	60.00	2.24	−0.308
GRA6	2087.33	10.90	37.78	112.97	−1.578
GRA7	2270.59	12.18	27.22	92.42	−1.944
MAG1	1981.98	3.71	22.94	60.63	−1.659
BSR4	1165.23	8.41	16.67	20.04	−1.067
CCp5A	2070.10	3.43	37.62	52.16	−0.281

Abbreviations: pI = isoelectric point, GRAVY = Grand Average of Hydropathy.

**Table 3:** Predicted linear B-cell epitopes and their antigenicity value obtained from the VaxiJen 2.0 server.

Peptide	VaxiJen 2.0 Antigenicity Value
SAG1	1.1163
SAG2A	1.2736
GRA1	1.2842
GRA2	1.0249
GRA3	1.2841
GRA5	0.7496
GRA6	0.3953
GRA7	0.6110
MAG1	1.2375
BSR4	1.4757
CCp5A	1.3231

Antigenicity Value: ≥0.5 probable ANTIGEN, <0.5 probable NOT ANTIGEN.

**Table 4:** ERRAT and PROVE analyses of three-dimensional modeled structures from whole molecules and peptides.

PROTEINS	Whole Molecule		Peptide	
	ERRAT	PROVE	ERRAT	PROVE
SAG1	90.7975	1.2%	100	5.0%
SAG2A	91.4286	4.5%	100	4.8%
GRA1	99.4413	3.3%	100	0%
GRA2	91.3295	3.2%	100	0%
GRA3	99.0566	1.9%	100	0%
GRA5	100	0.8%	100	0%
GRA6	92.2330	3.1%	100	0%
GRA7	97.7974	2.7%	100	4.0%
MAG1	92.0635	3.0%	100	5.0%
BSR4	97.1204	3.7%	100	0%
CCp5A	89.4467	4.2%	100	0%

## Data Availability

Data are contained within the article and Supplementary Materials.

## Supplementary Materials

The following supporting information can be downloaded at: https://www.mdpi.com/article/10.3390/ijms26104689/s1.
