# Supplementary material for: A Structural In Silico Analysis of Novel Epitopes from Toxoplasma gondii Proteins for the Serodiagnosis of Toxoplasmosis
Source: Int J Mol Sci. 2025 May 14;26(10):4689. doi: 10.3390/ijms26104689 (PMC12112391; doi:10.3390/ijms26104689)
Supplement: Supplementary file 1 [file ijms-26-04689-s001.zip › ijms-3538519-supplementary.pdf]

Supplementary Tables:

Table S1. Ramachandran plot analysis and overall G-Factor of modeled *T. gondii* proteins calculated by PROCHECK of PDB SUM.

Proteins	Regions				G - Factor
	Most allowed	Additional allowed	Generously allowed	Disallowed	
SAG1	87.6%	12.4%	0.0%	0.0%	0.54
SAG2A	84.6%	13.4%	0.7%	1.3%	0.10
GRA1	94.6%	5.4%	0.0%	0.0%	0.47
GRA2	90.3%	9.7%	0.0%	0.0%	0.40
GRA3	92.1%	6.8%	0.5%	0.5%	0.40
GRA5	98.1%	1.0%	1.0%	0.0%	0.58
GRA6	89.8%	9.1%	0.6%	0.6%	0.37
GRA7	88.8%	7.8%	1.5%	1.9%	0.26
MAG1	91.5%	7.0%	1.0%	0.5%	0.44
BSR4	86.1%	11.3%	1.1%	1.4%	0.24
CCp5A	83.2%	13.2%	1.3%	2.2%	0.10

Overall average G-factor value: values below -0.5 unusual, values below -1.0 highly unusual.

Table S2. Ramachandran plot analysis and overall average G-Factor of modeled *T. gondii* peptides calculated by PROCHECK of PDB SUM.

Proteins	Regions				G - Factor
	Most allowed	Additional allowed	Generously allowed	Disallowed	
SAG1	46.7%	53.3%	0.0%	0.0%	-0.32
SAG2A	81.8%	18.2%	0.0%	0.0%	-0.43
GRA1	72.7%	27.3%	0.0%	0.0%	-0.37
GRA2	84.6%	15.4%	0.0%	0.0%	-0.08
GRA3	90.9%	9.1%	0.0%	0.0%	-0.16
GRA5	75.0%	25.0%	0.0%	0.0%	-0.30
GRA6	92.9%	7.1%	0.0%	0.0%	-0.24
GRA7	64.3%	28.6%	7.1%	0.0%	-0,32
MAG1	85.7%	14.3%	0.0%	0.0%	-0.15
BSR4	87.5%	12.5%	0.0%	0.0%	-0.36
CCp5A	55.6%	44.4%	0.0%	0.0%	-0.44

Overall average G-factor value: values below -0.5 unusual, values below -1.0 highly unusual.

Supplementary Figures:

Figure S1

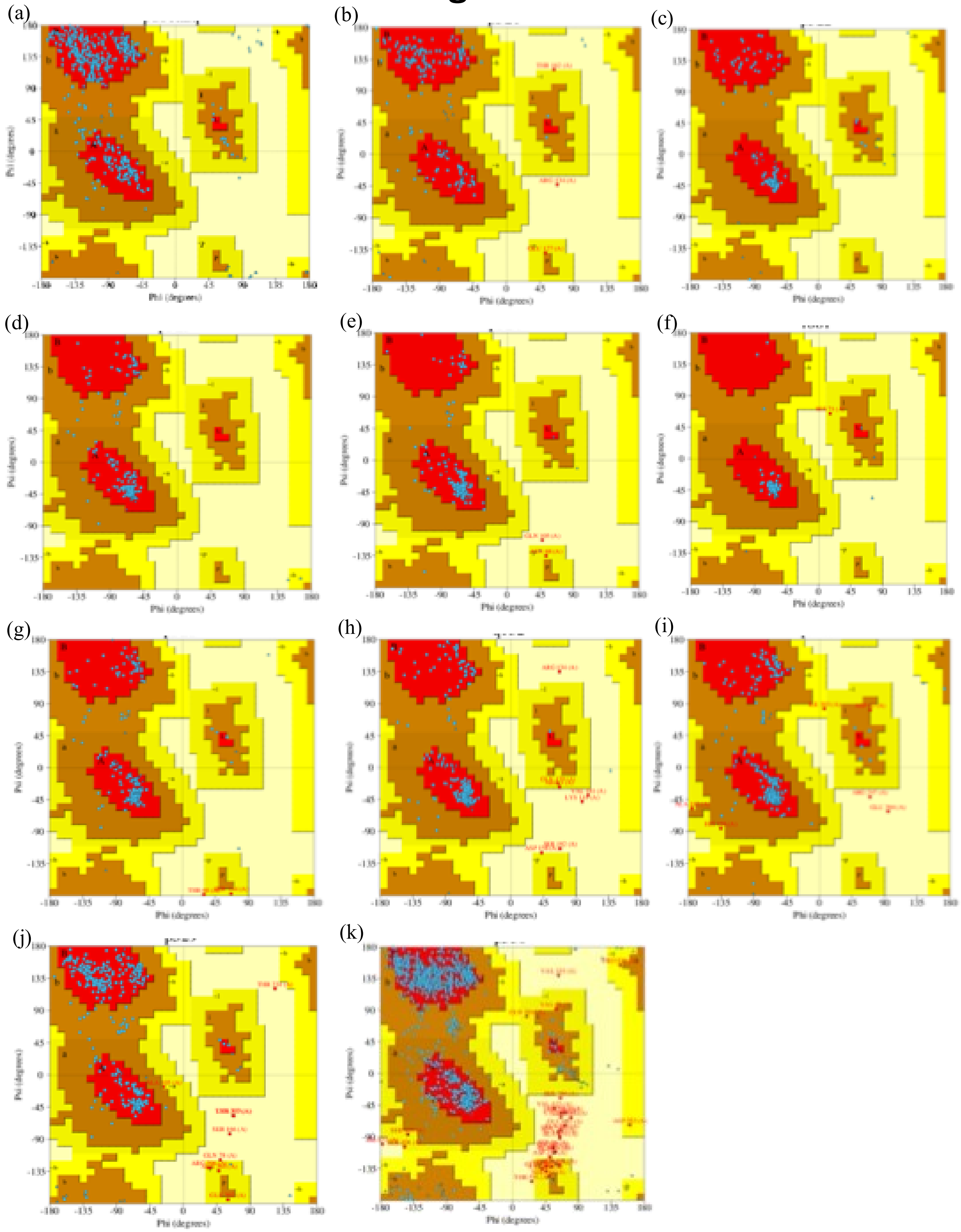

Figure S2

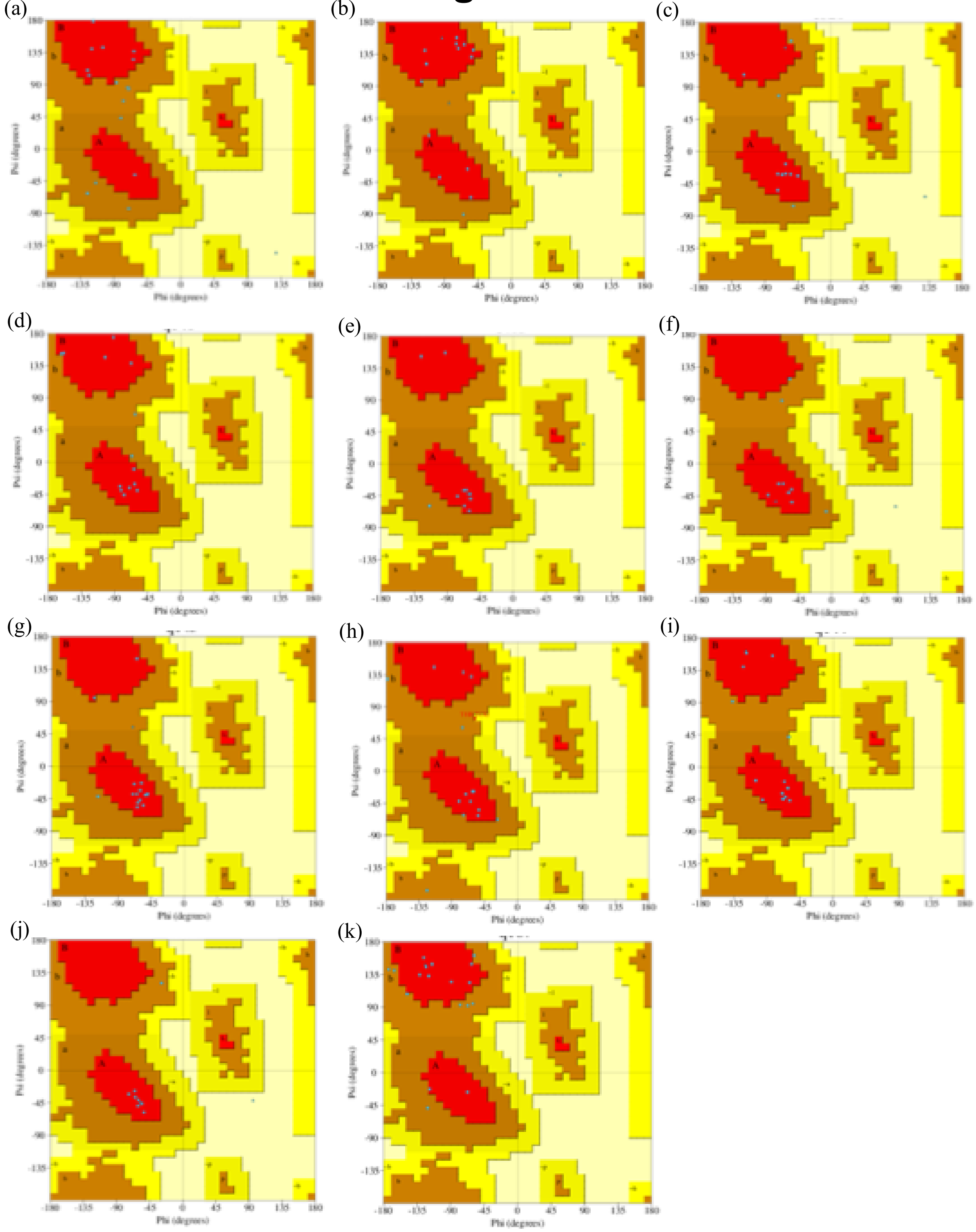
(a)
(b)
(c)
(d)
(e)
(f)
(g)
(h)
(i)
(j)
(k)
Psi (degrees)
Phi (degrees)
-180 -135 -90 -45 0 45 90 135 180
